# Depressive symptoms and myeloproliferative neoplasms: Understanding the confounding factor in a complex condition

**DOI:** 10.1002/cam4.3380

**Published:** 2020-09-25

**Authors:** Leslie Padrnos, Robyn Scherber, Holly Geyer, Blake T. Langlais, Amylou C. Dueck, Heidi E. Kosiorek, Zhenya Senyak, Matthew Clark, Michael Boxer, Mary Cotter, Claire Harrison, Cynthia Stonnington, Yonas Geda, Ruben Mesa

**Affiliations:** ^1^ Division of Hematology and Medical Oncology Mayo Clinic Scottsdale AZ USA; ^2^ Department of Hematology and Oncology UT Health San Antonio MD Anderson Cancer Center San Antonio, Portland Texas, Oregon USA; ^3^ Division of Hospital Medicine Mayo Clinic Scottsdale AZ USA; ^4^ Division of Biostatistics Mayo Clinic Scottsdale AZ USA; ^5^ MPN Forum MPN Research Foundation Chicago IL USA; ^6^ Department of Psychiatry and Psychology Mayo Clinic Rochester MN USA; ^7^ Arizona Oncology Tucson AZ USA; ^8^ Guy’s and St Thomas’ NHS Foundation Trust London United Kingdom; ^9^ MPN Voice London United Kingdom; ^10^ Department of Psychiatry and Psychology Mayo Clinic Scottsdale AZ USA; ^11^ Center for Bioelectronics and Biosensors Arizona State University Tempe AZ USA

**Keywords:** depression, essential thrombocythemia, myelofibrosis, myeloproliferative neoplasm, PHQ‐2, polycythemia vera, psychooncology, quality of life

## Abstract

**Background:**

Philadelphia chromosome negative myeloproliferative neoplasms (MPNs), including essential thrombocythemia, polycythemia vera, and myelofibrosis, have severe function‐limiting symptom burden that is experienced by the majority of patients. Previous studies have suggested that depression may be present in over a quarter of MPN patients, but to date no studies have evaluated the relationship between depression and other variables such as symptoms.

**Methods:**

A 70‐item internet based survey regarding fatigue and mood symptoms was developed by a multidisciplinary team of MPN investigators, patients and patient advocates including Patient Health Questionnaire and the Myeloproliferative Neoplasm Symptom Assessment Form was completed by over 1300 patients with MPN diagnosis.

**Results:**

There were 309 respondents (23%) with PHQ‐2 scores ≥ 3. In this analysis, we found worse systemic symptom burden in individuals reporting depressive symptoms.

**Conclusion:**

This analysis suggests the importance of depression in contributing to as well as confounding symptomatology in MPN patients, and suggests that this critical variable should also be addressed by clinicians and researchers alike when comprehensively assessing symptom burden etiologies.

## Graphical abstract

A survey of over 1300 patients with myeloproliferative neoplasms revealed 23% reported depressive symptoms and these symptoms were associated worse systemic symptom burden.

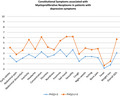

**Lay Summary**

Philadelphia chromosome negative myeloproliferative neoplasms (MPNs), including essential thrombocythemia, polycythemia vera, and myelofibrosis, have severe function‐limiting symptom burden that is experienced by the majority of patients. Previous studies have suggested that depression may be present in over a quarter of MPN patients, but to date no studies have evaluated the relationship between depression and other variables such as symptoms. We performed a survey of over 1300 MPN patients to investigate the relationship of depression to other disease variables and demographics. In this analysis, we found worse systemic symptom burden in individuals reporting depressive symptoms. This analysis suggests the importance of depression in contributing to as well as confounding symptomatology in MPN patients, and suggests that this critical variable should also be addressed by clinicians and researchers alike when comprehensively assessing symptom burden etiologies.

## 1 INTRODUCTION

Patients with a diagnosis of myeloproliferative neoplasms (MPN), including essential thrombocythemia (ET), polycythemia vera (PV), or myelofibrosis (MF) experience a spectrum of symptoms and sequela related to the stem cell disorder. The sequela can include laboratory abnormalities such as thrombocytosis in ET, erythrocytosis in PV or cytopenias in MF. In addition, patients can experience vascular complications of thrombosis or hemorrhage in ET and PV and splenomegaly in patients with PV or MF. In addition to the abnormalities demonstrated in laboratory tests and physical exam, patients with a diagnosis of MPN also experience constitutional symptoms including fatigue, pruritus, and night sweats.^1^ A large international survey of MPN patients identified fatigue as the most common and severe symptom consistently reported in this patient population^2^ and also revealed that nearly 25% of MPN respondents were at risk for depression as measured by the Patient Health Questionnaire‐2.

Myeloproliferative neoplasms are currently incurable for most patients. Thus, an MPN diagnosis denotes a lifelong cancer diagnosis and associated complications, increased interaction with the medical field, cancer directed therapies, and constitutional symptoms that can impact function and quality of life.

Depressive symptoms are commonly observed in patients with chronic medical conditions^3^ including cardiovascular disease,^4^ or rare chronic diseases.^5^ The relationship between depression and chronic medical disorders may be bi‐directional^6^ with each condition contributing to either the development or severity of the other. Depression can also impact survival within a patient population including diabetes,^7^ stroke,^8^ obesity,^9^ and cancer.^10^ Within myeloproliferative neoplasms, one international study reported 60% of patients with MPNs self‐reported “some level of depression” within the past one month, and one in five reported it impacted them.^11^

To date, characterizations of depression in MPNs have been limited and the long‐term psychological influence of this diagnosis unknown. We comprehensively describe the experience of depressive symptoms in patients with myeloproliferative neoplasms based on patient reported data from an internet based survey designed to investigate the experience of fatigue in MPN patients published previously by Scherber et al.^2^

## 2 MATERIALS AND METHODS

A 70‐item internet‐based survey regarding fatigue and mood symptoms was developed by a multidisciplinary team of MPN investigators, patients and patient advocates. This internet‐based survey focused predominantly on the symptom of fatigue has been previously published.^2^ The Mayo Clinic Institutional Review Board approved the study protocol. In February and March of 2014, the survey was promoted online in concert with multiple MPN‐related web sites including the MPN Forum, the MPN Research Foundation and the MPN Voice. It was hosted by the Mayo Clinic Survey Research Center. This survey was designed and advertised as a study of fatigue among MPN patients. All survey responses were anonymous and the data was captured and downloaded to secure servers at the Mayo Clinic Survey Research Center in Rochester, Minnesota. Participants self‐identified as patients with an MPN diagnosis able to understand written English. Participants did not receive compensation for completing the survey. Participants were asked to complete the survey only once and were required to complete a modified consent prior to accessing the survey.

### 2.1 Survey content

The survey included questions regarding patient demographics, disease type and features, lifestyle factors, co‐morbidities, MPN complications, MPN therapy, and symptoms. Patients were specifically asked to self‐report comorbid medical conditions including hypothyroidism, cytopenias, sleep disorders, obesity, and cardiopulmonary conditions. Mood disorder symptoms were assessed using the Profile of Mood States‐B (POMS‐B), Patient Health Questionnaire (PHQ‐2), and Mental Health Inventory (MHI‐5). The Patient Health Questionnaire‐2 (PHQ‐2)(Refer to Table 1) was completed by all respondents and utilized to assess depression. These depressive screening questions were chosen as they are able to indicate patients who are at high risk of depression, but do not diagnose depression. A PHQ‐2 score ≥ 3 was found to be sensitive (0.61) and highly specific (0.92) as a screening test for depression symptoms^12^ The Myeloproliferative Neoplasm Symptom Assessment Form (MPN‐SAF) was a validated measure given in conjunction with the Brief Fatigue Index (BFI) to assess MPN specific symptoms. The MPN‐SAF Total Symptom Score (MPN‐SAF TSS) is a compiled 10‐item assessment of the most representative MPN‐SAF symptoms. For individuals completing at least 6 of the 10 MPN‐SAF TSS items, the survey was scored by multiplying the average score across items by 10 to achieve a scaled score of 0‐100. The BFI was used as a standardized 10‐item measure of fatigue. Additionally, a focused and detailed assessment of fatigue characteristics and utilized nonpharmacological interventions was included.

**TABLE 1:** Patient health questionnaire‐2

Over the Past 2 wks, how often have you been bothered by any of the following problems?	Not at all	Several days	More than half the days	Nearly every day
Little interest or pleasure in doing things	0	1	2	3
Feeling down, depressed, or hopeless	0	1	2	3

### 2.2 Statistical analysis

Survey characteristics were compared between PHQ‐2 groups using t‐tests and Chi‐squared tests for continuous and discrete data, respectively. Comparisons of continuous data across MPN types were made using analysis of variance (ANOVA). P values less than 0.05 were considered statistically significant. SAS version 9.4 (Cary, NC) was used for the analysis.

## 3 RESULTS

### 3.1 Participants

The initial implementation of the survey collected a total of 1788 surveys. Of those providing consent to participate in the study, 1676 completed 10 or more survey questions. Of these, 1344 surveys reported a MPN subtype diagnosis and included complete responses needed to calculate the PHQ‐2. (Refer to Table 2). The self‐reported diagnoses included PV (550/1344, 40.9%), ET (445/1344, 33.1%), and MF (349/1344, 25.9%). The mean age was 59 years (standard deviation [SD] 11.8) with a female predominance (68%). The median PHQ‐2 score was 1 (range 0‐6.0) for the entire population (Refer to Table 3).

**TABLE 2:** Demographics

	PHQ ≥ 3 (N = 309)	PHQ < 3 (N = 1035	Total (N = 1344)	
Age, Mean (SD)	56.9 (12.2)	59.6 (11.6)	59.0 (11.8)	*P* = .0003
Gender, male	98(31.7%)	334 (32.3%)	432 (32.1%)	*P* = .85
Race (White)	294 (95.5%)	996 (96.4%)	1290 (96.2%)	*P* = .53
Education				
Middle school	6 (1.9%)	11 (1.1%)	17 (1.3%)	*P* < .0001
High School	86 (27.9%)	233 (22.7%)	319 (23.9%)	
Undergrad	149 (48.4%)	405 (39.4%)	554 (41.5%)	
Master's or advanced degree	58 (18.8%)	294 (28.6%)	352 (26.4%)	
Doctorate	9 (2.9%)	84 (8.2%)	93 (7.0%)	
MPN Diagnosis				
ET Diagnosis	107 (34.6%)	338 (32.7%)	445 (33.1%)	*P* = .78
PV Diagnosis	122 (39.5%)	428 (41.4%)	550 (40.9%)	
MF Diagnosis	80 (25.9%)	269 (26%)	349 (26%)	
Time since first MPN diagnosis				
<1 y ago	36 (11.7%)	73 (7.1%)	109 (8.1%)	*P* = .08
1‐3 ys ago	66 (21.5%)	204 (19.97%)	270 (20.1%)	
>3 y ago	205 (66.8%)	756 (73.2%)	961 (71.7%)	
MPN characteristics				
Prior splenectomy (y)	12(3.9%)	24 (2.3%)	36 (2.7%)	*P* = .13
Thrombosis	72 (23.4%)	171 (16.6%)	243 (18.2%)	*P* = .0071
JAK2 V617F mutation				
Positive	158 (66.4%)	605(73.6%)	763 (72%)	*P* = .0161
Negative	80(33.6%)	217 (26.4%)	297 (28%)	
Missing	213	71	284)	
Cytopenias				
Anemia	163(53.3%)	478 (46.6%)	641 (48.1%)	*P* = .04
Received any blood product transfusion	43 (14.1%)	125 (12.2%)	168 (12.6%)	*P* = .37
Received any blood product transfusion in prior 6 mo	16 (5.17%)	57 (5.5%)	73 (5.4%)	*P* = .25

**TABLE 3:** Reports of PHQ scoring

PHQ2 Score	PHQ2 ≥ 3 (N = 309)	PHQ2 < 3 (N = 1035)	PHQ2 ≥ 3 ET (N = 107)	PHQ2 ≥ 3 PV (N = 122)	PHQ2 ≥ 3 MF (N = 80)	Total (N = 1344)
0	0 (0.0%)	448 (43.3%)	0 (0.0%)	0 (0.0%)	0 (0.0%)	448 (43.3%)
1	0 (0.0%)	247 (23.9%)	0 (0.0%)	0 (0.0%)	0 (0.0%)	247 (23.9%)
2	0 (0.0%)	340 (32.9%)	0 (0.0%)	0 (0.0%)	0 (0.0%)	340 (32.9%)
3	116 (37.5%)	0 (0.0%)	35(32.7%)	43(35.2%)	38(47.5%)	116 (37.5%)
4	88 (28.5%)	0 (0.0%)	37(34.6%)	37(30.3%)	14(17.5%)	88 (28.5%)
5	53 (17.2%)	0 (0.0%)	13(12.1%)	24(19.7%)	16(20%)	53 (17.2%)
6	52 (16.8%)	0 (0.0%)	22(20.6%)	18(14.8%)	12(15%)	52 (16.8%)

The majority of patients identified their race as White (96%), followed by multiracial (1.6%), Asian (1.4%), or Black or African American (0.9%). The majority of patients were from the United States of America (65%). Comorbidities included hypothyroidism (N = 175, 13%), restless leg syndrome (N = 95, 7.1%), heart or kidney failure (N = 84, 6.3%), obstructive sleep apnea (N = 52, 3.9%), rheumatologic disease (N = 51, 3.8%), and fibromyalgia (N = 47, 3.5%). With regards to restless leg syndrome and MPN subtype, it was more prevalent in the PV group (52/550, 9.5%) and less common in ET (22/445, 5.0%) or MF (21/349, 6.0%). Anemia (N = 641, 48%), splenomegaly (N = 526, 40.8%), and thrombosis (N = 243, 18.2%) were common MPN physiologic complications reported by participants. Most respondents, (N = 1113, 85%) had received MPN‐directed pharmacological treatments, while (N = 644) 54% had undergone phlebotomy and (N = 161) 14.6% reported use of transfusions. The most common medications were aspirin (81.8%), hydroxyurea (75.8%), interferon (24.3%), anagrelide (24.3%), Ruxolitinib/JAK2 inhibitor (15.2%), and anticoagulant (10.8%).Seventy‐two percent of respondents were JAK2 mutation positive. The average MPN‐Total Symptom Score (TSS) was 28.3 (average for patients with PV was 29.2, average for patients with ET was 28.6, the average for patients with MF was 26.3).

Approximately 75% of respondents reported the highest level of education completed as an undergraduate degree or higher (Undergrad 41.5%, Master's degree 26.4%, Doctorate degree 7.0%).

### 3.2 Depressive symptoms and associations

#### 3.2.1 Patient characteristics

There were 309 respondents (23%) with PHQ‐2 scores ≥ 3. Characteristics grouped by PHQ‐2 scores ≥ 3 and < 3 are shown in Table 3. Of the 309 respondents who reported a spectrum of PHQ‐2 ≥ 3, the PHQ‐2 scores ranged from 3 (38%), 4 (29%), 5 (17%) and 6 (17%). MPN subtype and gender did not differ between PHQ‐2 scores ≥ 3 and < 3 (*P* = .78 and *P* = .85, respectively). A PHQ‐2 score of ≥ 3 was associated with younger age (mean 56.9 (SD 12.2), vs 59.6 (SD 11.6) years; *P* = .003).

Higher education levels were associated with lower rates of depressive symptoms (Table 2, *P* < .0001), with Master's and Doctorate degrees less frequently reporting PHQ‐2 ≥ 3 (master's 18.8% vs 28.6%; doctorate 2.9% vs 8.2%).

An increase in depressive symptoms was associated with increased emotional stress (66.4% vs 45.0%, *P *=< .0001) and new sleep disturbances (33.7% vs 19.9%, *P* < .0001).

Participants with PHQ‐2 ≥ 3 were more likely to smoke cigarettes (10.9% vs 5%, *P* = .0003), actively use prescription pain medication (24.4% vs 10.6%, *P* < .0001), antidepressants (31.3% vs 11.9%, *P* < .0001), and anxiety medications (15.4% vs 8.6%, *P* = .0005), and were less likely to report regular exercise use (*P* < .0001).

### 3.3 MPN diagnosis

The proportions of patients with PHQ‐2 ≥ 3 were similar across MPN types (ET 34.6%, PV 39.5%, MF 25%, *P* = .78). The frequency of patients reporting previous MPN directed treatments was similar between the two PHQ groups (phlebotomy 55.16% PHQ ≥ 3, *P* = .3691; medications 86.9% PHQ ≥ 3, *P* = .3365; JAK inhibitor use specifically 16.2% PHQ ≥ 3, *P* = .6432; and transfusions 17.3% PHQ ≥ 3, *P* = .1833) (Refer to Tables 4 and 5). A PHQ‐2 ≥ 3 was associated with a lower presence of JAK V617F mutation (66.4% PHQ ≥ 3% vs 73.6% PHQ < 3, *P* = .0291), however, a thrombosis history was associated with higher rates of depressive symptoms (23.4% PHQ ≥ 3, compared to 16.6% PHQ < 3, *P* = .007).

**TABLE 4:** Prior or current mpn‐directed treatment

	PHQ ≥ 3 (N = 309)	PHQ < 3 (N = 1035)	TOTAL (N = 1344)	
Allogeneic stem cell transplant	3 (1.2%)	12 (1.4%)	15 (1.4%)	NS
Phlebotomy	143 (51.6%)	501 (54.7%)	644 (54.0%)	NS
Medications	266 (86.9%)	847 (84.7%)	1113 (85.2%)	NS
Radiation	4 (1.6%)	6 (0.7%)	10 (1.0%)	NS
Transfusion	44 (17.3%)	117 (13.9%)	161 (14.7%)	NS

Note

*P*‐value> .05.

Abbreviation: NS, non‐significant.

**TABLE 5:** Prior of current MPN‐directed pharmacological treatment

	PHQ ≥ 3 (N = 309)	PHQ < 3 (N = 1035)	Total (N = 1344)	
Hydroxyurea	210 (78.9%)	632 (74.6%)	842 (75.7%)	NS
Aspirin	222 (83.5%)	688 (81.2%)	910 (81.8%)	NS
Interferon	54 (20.3%)	212 (25%)	266 (23.9%)	NS
Anagrelide	75 (28.2%)	196 (23.1%)	271 (24.3%)	NS
Anticoagulants	26 (9.8%)	94 (11.1%)	120 (10.8%)	NS
Thalidomide/Pomalidomide	5 (1.9%)	19 (2.2%)	24 (2.2%)	NS
Lenalidomide	4 (1.5%)	16 (1.9%)	20 (1.8%)	NS
Busulphan	8 (3%)	22 (2.6%)	30 (2.7%)	NS
Prednisone	17 (6.4%)	51 (6.0%)	68(6.1%)	NS
Danazol	3 (1.1%)	10 (1.2%)	13 (1.2%)	NS
Ruxolitinib or other JAK2 inhibitor	43 (16.2%)	127 (15.0%)	170 (15.3%)	NS

Note

*P*‐value> .05.

Abbreviation: NS, non‐significant.

While PHQ‐2 ≥ 3 was associated with reports of anemia (53% vs 46%, *P* = .04), there was no difference in reported depressive symptoms and anemia‐directed therapies including recent transfusions (prior 6 months) (*P* = .25), erythropoietin injections (*P* = .3), or iron supplementation (*P* = .9).

### 3.4 MPN related symptoms

Worse depressive symptoms (PHQ‐2 ≥ 3) were associated with higher MPN‐SAF Total Symptom Score (mean 40.8 (SD 16.7) vs 24.6(SD 15.9); *P* < .001), higher worst fatigue score (mean 7.8 (SD 1.9) vs 5.8 (SD 2.7); *P* < .001), and worse overall quality of life (mean 5.7(SD 2.1) vs 3.1(2.2); *P* < .0001).

**FIGURE 1:**
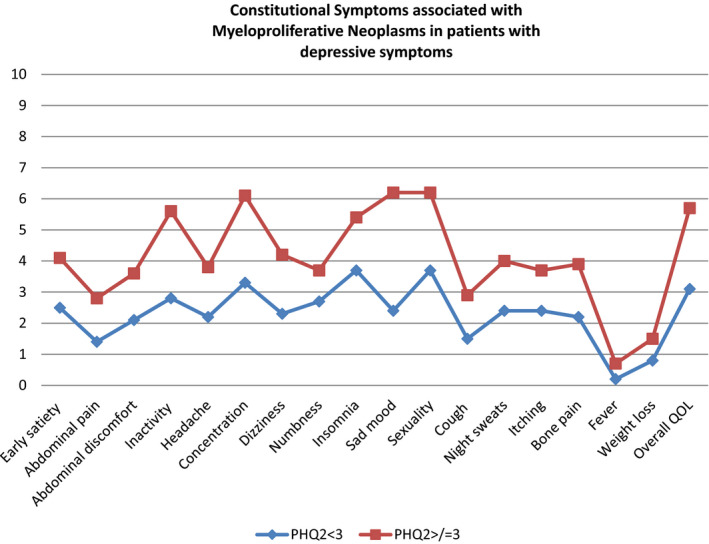
Constitutional symptoms associated with depressive symptoms

Each of the individual symptoms assessed in the MPN‐SAF were rated significantly higher (worse) in the population of respondents reporting a PHQ‐2 ≥ 3 (all *P* < .0001). See Figure 1.

The timing of fatigue was distinct between those with PHQ‐2 ≥ 3 and those with PHQ‐2 < 3. Individuals with PHQ‐2 ≥ 3 were more likely to experience fatigue all day (42.7% vs 17.5%, *P* < .0001) while those with PHQ‐2 < 3 were more likely to experience fatigue in the evening (PHQ2‐<3 47% vs 35%, *P* < .001). Progression of fatigue over the prior 6 months was associated with higher rates of depressive symptoms (very much worse (13% vs 4.2%) and moderately worse (29% vs 12%), *P* < .0001).

Patients endorsed a variety of management options to address fatigue. Patients with worse PHQ‐2 scores were more likely to try to manage fatigue by setting priorities (79.7% vs 73.9%, *P* < .05), postponing non‐essential activities (82.9% vs 71.8%, *P* = .0001), napping (76% vs 68.3%, *P* < .05), while patients with PHQ‐2 < 3 scores were more likely to use exercise (77.1% vs 62.5%, *P* < .0001) and volunteering (33.4% vs 24.6%, *P* < .005).

## 4 CONCLUSION

In this large international survey of patients with MPNs, nearly a quarter of individuals reported significant depressive symptoms. To the best of our knowledge, this is the most comprehensive assessment and analysis of depressive symptoms impacting this patient population at risk for physiologic and constitutional symptoms related to underlying hematologic malignancy.

Individuals with significant depressive symptoms have worse systemic symptoms based on the MPN‐TSS. Higher levels of depressive symptoms were associated with significantly higher levels of systemic symptoms in PV and MF, and all but two constitutional symptoms in ET (numbness, itching). Higher levels of depressive symptoms were associated with fatigue experienced throughout the day, though the cause or effect of this temporal relationship is not known. The presence of constitutional symptoms impact prognostic scoring for myelofibrosis and is a key feature in therapy response in JAK2 inhibition, indicating the importance of the presence and mitigation of these physiologic experiences. The association of MPN constitutional symptoms with prominent depressive symptoms suggests that heavy constitutional symptom burden leads to depressive symptoms and addressing the MPN symptom severity, through pharmacological or nonpharmacological therapies, may improve symptoms of depression. In contrast, or in addition, prominent depressive symptoms may worsen the severity of systemic symptoms experienced by MPN patients and utilizing psychological therapy to address the underlying mood disorder may lessen MPN symptoms. These finding are consistent with a prior study of 117 MPN patients demonstrating an association between physical symptom burden and psychologic symptoms, predominantly depressive symptoms as assessed by the Distress Thermometer and Problem List (DT&PL) and Hospital Anxiety and Depression Scale (HADS).^13^

Further understanding regarding the cause‐and‐effect of depression and the constitutional symptoms in MPN is needed.

One study of 107 patients with a spectrum of hematologic malignancy diagnoses (predominantly lymphoma, acute leukemia and plasma cell dyscrasias) reported that 17% had high levels of depression reported on the Hospital Anxiety and Depression Scale (HADS), and that a more recent diagnosis was associated with increased level of intrusive thought symptoms.^14^ In a study of 304 Australian patients with a diagnosis of a hematologic cancer, 17% of patients reported depression and 27% reported anxiety with associated factors of relocation for treatment and smoking history, but no association for age, hematologic malignancy subtype, employment status or cancer treatment.^15^ A recent study assessing depressive symptoms utilizing of the Patient Health Questionnaire‐9 (PHQ‐9) in 104 patients with hematologic malignancy preparing to receive initial intravenous chemotherapy revealed that 15% reported significant depressive symptoms.^16^ There was no association between the PHQ‐9 score and medication use, number of visit needed for malignancy management, age, sex, or marital status. As patients with myeloproliferative neoplasms do not traditionally utilize intravenous chemotherapy for treatment, no MPN patients were included in this study. Research suggests that psychosocial factors impact cancer development and experience differently based on subtype, with increased stress or emotional distress associated with increased cancer incidence and mortality.^10^ Decreased cancer survival has been associated with psychosocial factors impacting hematopoietic cancers, breast, lung, and head and neck cancer. Another study showed emotional non‐expression was associated with reduced survival in localized melanoma.^17^

Understanding that the burden of depression impacts somatic health, and specifically appears associated with risk of onset and progression of cardiovascular disease^18^ is relevant to MPN patients as cardiovascular disease is a major risk to mortality and morbidity.^19^ The possible contributing mechanisms linking depression to cardiovascular disease include unhealthy lifestyle behaviors including smoking and physical inactivity as well as metabolic and immune‐inflammatory dysregulation on a cellular level. In depression, the hypothalamic‐pituitary‐adrenal axis can be hyperactive and/or dysregulated which leads to multiple changes within the central nervous system including atrophy of brain cells, reduced neurogenesis and altered neurotransmitter signaling while inflammatory dysregulation has also been associated with increased depressive symptoms with higher levels of certain cytokines, including IL‐6 and tumor necrosis factor alpha (TNF‐alpha) noted in depressed patients compared to controls^20^ Studies assessing cytokine levels in individuals with MPNs have also demonstrated increased circulating inflammatory cytokines of IL‐6, TNF‐alpha in addition to a variety of other elevated cytokine levels.^21^

Our study, exclusively evaluating symptoms in patients with MPN diagnosis, revealed a risk of depressive symptoms in approximately 20% of patients which is consistent with the reports of depressive symptoms in other hematologic malignancies. This suggests that patients with MPN diagnosis, regardless of treatment type, have a similar risk of depressive symptoms as individuals with high risk hematologic malignancies undergoing aggressive, often intravenous, treatment.

Limitations for this study include the use of the PHQ‐2 as a screening test, which can serve as a first step in assessing patients for depressive symptoms but does not diagnose depression. In order to confirm clinical depression and evaluate the need for treatment a patient would need to meet with a physician in person. In addition, the respondents were primarily white and highly educated patients from the United States of America. Understanding the frequency and distribution of depressive symptoms in patients with various racial, socioeconomic, ethnic, cultural backgrounds would be important and further investigation within this space is warranted. Other limitations due to the cross‐sectional study and one‐time point assessed for all patients include lack of understanding regarding the timing of attempted mood related therapies, such as counseling or anti‐depressants, and the efficacy with regards to impact on depressive or MPN symptoms. This study, due to a one‐time assessment of symptoms, does not allow for understanding if there are certain phenotypes of depressive symptoms in patients with MPN predisposing some individuals to a chronic risk of experience, or an evolution of symptoms over time. Further research is necessary and should include a longitudinal study of patients regarding their MPN diagnosis, treatment, constitutional symptom burden, and depressive symptom experience.

Mental well‐being is a critical feature to living a healthy and active life. Significant depressive symptoms are not uncommon in patients with MPNs. The mood disturbances were not associated with MPN‐related therapy options but were associated with worse systemic symptom burden. This indicates the depressive symptomatology may be intrinsic to the condition, in addition to a result of treatment side effects. Depression for MPN patients is due to more than just the psychological stress of a worrisome diagnosis. The lifetime of uncontrolled symptom burden and disease features have been demonstrated to impair significant social, role, and physical functioning.^11^ Additionally, environmental factors such as financial stressors can significantly contribute to depression. Depression may be a result of neurological impairment from macrovascular complications such as thrombosis or microvascular complications with the central nervous system. In addition, inflammation has been associated with both myeloproliferative neoplasms^21^ and depression.^22^

It is also important to note that certain treatments in MPNs are contraindicated in patients with depression. Interferon, the only treatment for PV and ET that has demonstrated complete molecular remission in a minority of patients, is contraindicated in patients with depression. In previous studies, interferon use was significantly associated with depression in patients with malignancy, infection or autoimmune conditions.^23^

Based on these findings, the clinical suspicion for depression in MPN patients should be high, and screening for depression should be routine in this patient population. Little data exists regarding the efficacy of traditional treatments in this population. In our study, we have demonstrated that symptoms have an integral and highly associated relationship with depression, and thus, alleviation of symptom burden as a therapeutic target may be warranted in order to aid depression. It may also be true that treating the symptoms of depression in patients with MPN may lead to lessened symptom burden. Interventions that promote mindfulness and stress reduction may also be warranted, particularly ones that can be paired with traditional therapy. Our group has demonstrated promising data in our previous investigations including yoga, mindfulness, and ACT therapy. Future investigations into the therapeutic alleviation of depression or depressive symptoms in myeloproliferative neoplasms are warranted.

## CONFLICTS OF INTEREST

Authors LP, HG, BL, AD, HK, ZS, MC, MB, CH, CS, YG report no conflicts of interest. Author RS reports Honorarium with Incyte, CTI Biopharma and Celgene. Author M.Clark reports Consultant, Roche GmbH Diabetes. Author RM reports Consultant for Novartis, Sierra Onc, La Jolla, Pharma. Research Support from Celgene, Incyte, Abbvie, Samus, Genetech, Promedior, CTI.

## AUTHOR CONTRIBUTIONS

Leslie Padrnos contributed to conceptualization, methodology, project administration, writing—original draft, and writing—review and editing. Robyn Scherber contributed to conceptualization, methodology writing, review and editing. Holly Geyer contributed to writing, review and editing. Blake Langlais contributed to data curation, formal analysis, review and editing. Amylou Dueck and Heidi Kosiorek contributed to formal analysis, review and editing. Zhenya Senyak contributed to resources, writing—review and editing. Matthew Clark, Cynthia Stonnington, and Yonas Geda contributed to supervision, writing—review and editing. Michael Boxer and Mary Cotter contributed to resources, writing—review and editing. Claire Harrison contributed to resources, methodology, writing—review and editing. Ruben Mesa contributed to conceptualization, resources, funding acquisition, project administration, supervision, writing—review and editing.
